# Identification of Two Novel Compound Heterozygous *EIF2AK3* Mutations Underlying Wolcott–Rallison Syndrome in a Chinese Family

**DOI:** 10.3389/fped.2021.679646

**Published:** 2021-05-26

**Authors:** Na Zhao, Yanling Yang, Ping Li, Qiuhong Xiong, Han Xiao, Changxin Wu

**Affiliations:** ^1^Institutes of Biomedical Sciences, Key Laboratory of Chemical Biology and Molecular Engineering of National Ministry of Education, Shanxi University, Taiyuan, China; ^2^Department of Pediatrics, Peking University First Hospital, Beijing, China

**Keywords:** WRS, EIF2AK3, PERK, diabetes, variation

## Abstract

**Objective:** Wolcott–Rallison syndrome is a rare autosomal recessive inheritance disorder caused by the defectiveness of eukaryotic translation initiation factor 2 alpha kinase 3 (*EIF2AK3*), which encodes the PKR-like endoplasmic reticulum kinase (PERK). Defect in *EIF2AK3* results in a permanent diabetes in early infancy or newborn period, a tendency to develop skeletal fractures and other associated disorders such as severe liver and renal dysfunction, and central hypothyroidism. Two patients with Wolcott–Rallison syndrome-like manifestations in a Chinese family and family members were genetically analyzed to identify if any variations that occurred in *EIF2AK3*, which may cause Wolcott–Rallison syndrome.

**Methods:** Whole-exome sequencing (WES) was performed to identify genetic variations, and Sanger sequencing was conducted to verify the identified variations in the family members with Wolcott–Rallison syndrome (WRS) clinical manifestations. Several bioinformatics tools were employed to predict the effect of *EIF2AK3* variations on the protein function. The impact on PERK protein was analyzed by sequential analysis and evolution conservation study.

**Results:** Two novel *EIF2AK3* heterozygous single base variations (c.2818C>T and c.2980G>C) were detected in the proband. PERK has two functional domains: one is regulatory domain (aa 1–576), and the other is catalytic domain (aa 577–1,115). Both variations are missense mutations and locate in catalytic domain of PERK; c.2818C>T resulted in a residue substitution of proline for serine at amino acid site 940 (p.Pro940Ser), and variation c.2980G>C caused an amino acid change at position 994 from glutamic acid to glutamine (p.Glu994Gln). These novel missense variations may affect the physiological functions of PERK protein.

**Conclusions:** Two novel compound heterozygous *EIF2AK3* variations (c.2818C>T, p.Pro940Ser and c.2980G>C, p.Glu994Gln) were found in a Chinese family. The identification of the variations and verification of their pathogenicity extended the variation spectrum of *EIF2AK3* variations causing Wolcott–Rallison syndrome and enriched valuable information for precise medical intervention for Wolcott–Rallison syndrome in China.

## Introduction

Wolcott–Rallison syndrome (WRS, OMIM: 226980) is caused by an autosomal eukaryotic translation initiation factor 2-a kinase 3 (*EIF2AK3*) deficiency, associated with permanent diabetes and skeletal dysplasia in newborn period or early infancy, hepatic dysfunction, and growth retardation, which was first reported in 1972 by Wolcott and Rallison (1–3). As an autosomal recessive inheritance disorder, WRS is more common in consanguineous families. In 2000, *EIF2AK3* was confirmed to be the disease-causing gene of Wolcott–Rallison syndrome (2). So far, almost 100 *EIF2AK3* variations causing WRS have been reported in the Human Gene Mutation Database (HGMD, http://www.hgmd.org/;2019.9).

The *EIF2AK3* gene (NC_000002, NM_004836.7) is located in chromosome 2p11.2, containing 17 exons, and encodes PKR-like endoplasmic reticulum kinase (PERK) (2). PERK is a transmembrane enzyme, which is highly expressed in both pancreatic beta cells and bone tissue. It is essential for normal fetal and early beta cell proliferation, differentiation, proinsulin processing, and stimulation of bone development (4). PERK acts as a major physiological effector of the unfolded protein response (UPR) following endoplasmic reticulum (ER) stress (5, 6). Once activated by the accumulation of misfolded proteins during ER stress, PERK phosphorylates the alpha subunit of the eukaryotic initiation factor-2 (EIF2A), thereby reducing the synthesis of misfolded proteins and increasing the expression of activating transcription factor 4 (ATF4), which regulates autophagy, amino acid metabolism, oxidative stress, and apoptosis. Loss-of-function mutations in the *EIF2AK3* gene decrease the ability of the ER to cope with stress, which results in loss of functional coordination among PERK-dependent ER chaperones responsible for controlling protein synthesis and proinsulin aggregation. These effects lead to β-cell defects and cell apoptosis, which results in permanent neonatal diabetes and epiphyseal dysplasia. Long-term regular insulin therapy has been demonstrated to improve the survival rates of WRS patients. Organ transplantation is a treatment for WRS; so far, three cases with organ transplantation have been reported (7–9), and these three patients were fully physically and socially rehabilitated.

In our study, we found compound heterozygous variations in *EIF2AK3*, which cause WRS in two children from non-consanguineous parents.

## Methods

### Patients' Information

This study was approved by the Ethics Committees of Shanxi University, and written informed consent was obtained from all the members of the family. The patient has been subjected to clinical and physical examinations, and all the medical records were reviewed and evaluated. The proband was diagnosed with diabetes at The Affiliated Hospital of Xuzhou Medical University (Xuzhou, China) in 2009. In 2017, the proband was diagnosed with WRS in The First Hospital of Peking University (Beijing, China).

### Molecular Genetic Studies

Whole-exome sequencing and validation by PCR were performed as described in our previous studies (10). The blood samples used for whole-exome sequencing were obtained from patients II-2 and II-3; the blood samples used for PCR and Sanger sequencing were obtained from family members I-1, I-2, and II-2. Variants were functionally annotated and filtered using our cloud-based rare disease NGS analysis platform (https://www.gene.ac/) in which analyses were performed by comparison with public databases [dbSNP, OMIM, ESP, Clivar, 1000 Genomes (11), and ExAc (12)] and HGMD Professional database.

The primer pairs for detecting the mutations by Sanger sequencing were as follows: Fw, 5′-AGTACTTGTCTGGCAC-3′; Rv, 5′-GGAACACTACTGCCAGTT-3′.

### Bioinformatics Analysis

Mutation Taster, PolyPhen-2, PROVEN, and SIFT were employed for the pathogenicity prediction of the variations in *EIF2AK3* gene. Evolutionary conservation of the altered amino acid residue was compared across different species.

## Results

### Clinical Manifestations

The proband (II-3) was first diagnosed as diabetes when she was 1-year old. Later on, she developed emesis and hypersomnia at 4 years old; clinical examination showed that the blood C-peptide levels was 0.01 nmol/L, which was markedly lower than the normal range (0.40 ± 0.20 nmol/L). The patient's blood glucose level was 2.07 mmol/L (normal range, 3.9–6.1 mmol/L). The urinalysis showed that the patient had glucosuria, ketonuria, and proteinuria. After receiving insulin rejection to control the blood glucose and symptomatic treatment, the patient was discharge 1 day later. At the age of 8, she was admitted with liver dysfunction and pneumonia, the clinical examination revealed a blood glucose of 13.7 mmol/L, alanine aminotransferase (ALT) of 852 U/L (normal range, 7–40 U/L), aspartate aminotransferase (AST) of 750 U/L (normal range, 5–35 U/L), albumin of 31.0 g/L (normal range, 35–55 g/L), prothrombin time of 20 s (normal range, 9–13 s), lactic dehydrogenase (LDH) of 2,150 U/L (normal range, 120–250 U/L). The X-ray showed that the patient had lobar pneumonia in the upper right lobe. After treatment with diammonium glycyrrhizinate, mezlocillin, and insulin, the patient was discharged 16 days later. The patient had no apparent skeletal deformities. The sister of the proband (II-2) was first diagnosed with diabetes and diabetic ketoacidosis at 4 years old and was then on insulin therapy. When the sister was 8 years old, she developed infection of the upper respiratory tract without apparent skeletal deformities. The brother (II-1) of the proband died of diabetes when he was 17 years old. The parents of the proband have no symptom of diabetes.

### Identification of *EIF2AK3* Compound Heterozygous Variations

**Figure 1:**
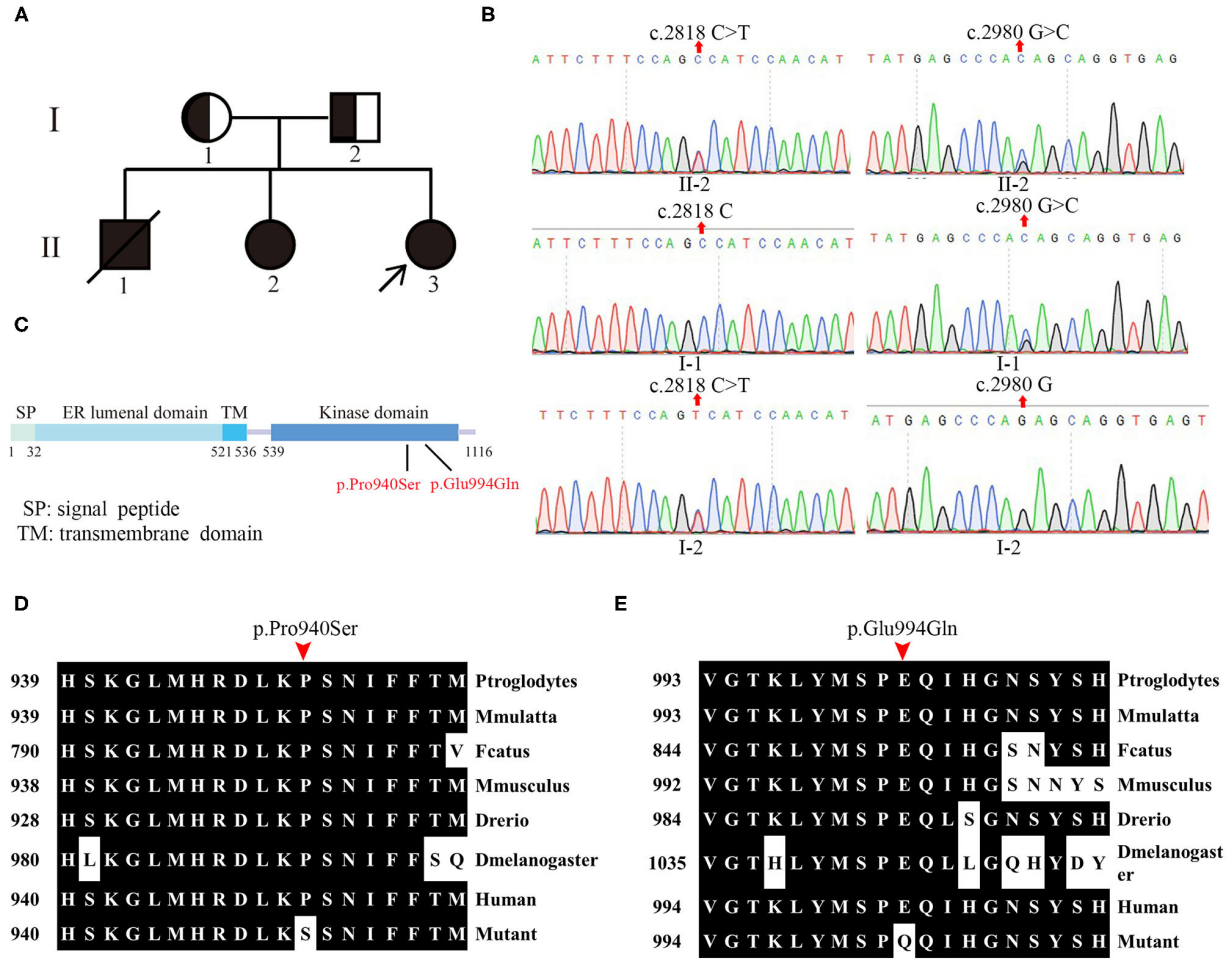
Pedigree and genotype of patients. **(A)** Pedigree of patients from the Chinese Wolcott–Rallison syndrome (WRS) family. The families involved in this study. Squares indicate male, circles indicate female, blackened symbols denote affected individuals, and half-blacked symbols denote the mutation carriers. The proband is indicated by arrows (↗). **(B)** The sanger sequencing analysis of the family. **(C)** Linear schematic of the protein structure of wide-type PERK. **(D,E)** Evolutionary conservation of amino acid residues altered by the mutations across different species.

The pedigree of the family is shown in Figure 1A. By whole-exome sequencing, two potential mutations (c.2818C>T and c.2980G>C) were detected in *EIF2AK3* gene from the proband, which was reported to associate with WRS. Subsequently, these two compound heterozygous variations were verified by Sanger sequencing among her family.

The novel heterozygous single base substitution c.2818C>T in exon 13 of *EIF2AK3* gene was detected in the mother (I-1) of the proband but not in her father, which is predicted to cause an amino acid change at position 940 (Figure 1B). The other novel heterozygous single base substitution c.2980G>C in exon 13 was detected in the father (I-2) of the proband but not in her mother, which is predicted to cause an amino acid change at position 994 (Figure 1B). Both variations were predicted to be causative factors of the disease by Mutation Taster, PolyPhen-2, PROVEAN, and SIFT.

The results from whole-exome sequencing and Sanger sequencing of her sister (II-2) showed that these two novel mutations from the proband also existed in her sister (Figure 1B).

### Molecular Analysis

These two novel mutations were both in the kinase domain of the PERK protein, which indicates that these mutations likely lead to WRS (Figure 1C). Evolution conservation analysis of amino acid showed that the impaired amino acid residues Pro940 and Glu994 in PERK protein were highly evolutionary conserved among different species (Figures 1D,E). Bioinformatic and biochemical assessment of the effect of the variations on the functions of PERK shows that both missense variations are located at the catalytic domain, a residue substation of Proline/P with Serine/S at the catalytic domain conserved region of PERK, which has the potential for phosphorylation, therefore affecting its functions. Glutamine replacing glutamic acid introduces charge change from negative and acidic residue to neutral residue, which may also bring about the functional change of PERK. In conclusion, the two novel compound heterozygous missense variations were identified by WES and verified further by Sanger sequencing in the *EIF2AK3* gene causing WRS in members of the family.

## Discussion

We identified two novel compound heterozygous variations in the *EIF2AK3* gene in a family, which included three patients with Wolcott–Rallison syndrome (WRS). So far, there are four families with WRS reported without homozygous variations in China (13). It seems that WRS has a low morbidity in China. Until April 2020, 98 variations were reported as the disease causative factors of WRS in HGMD, suggesting that the incidence of WRS is very low worldwide.

From previous reports, 96 variations in *EIF2AK3* involving 176 patients were identified for analysis in this study. Among the patients, there were 58 female patients, 68 male patients, and 50 patients without gender identification. Among the patients who indicated their gender, male patients accounted for 53.9%, and female patients accounted for 46.1%. It seems that there is no significant difference between genders on the morbidity of permanent neonatal diabetes mellitus (PNDM) caused by the variations in *EIF2AK3*. Functional analysis was performed in only nine variations. In addition to diabetes, the major symptoms of WRS are skeletal dysplasia and living and kidney dysfunction.

From Table 1, we find that among the clinical symptoms, skeletal dysfunction is the most common symptom in patients with WRS besides diabetes. The incidence of skeletal dysfunction is nearly 52.08% in this analysis. Base pair substitution mutation is the most common mutation type among all the variations. The percentage of small deletion that suffers both liver and kidney damage is the largest among all the mutation types. The patients with small deletion always suffer more symptoms. For example, the patients with variations c.1475-1476del (8), c.1567-1570del (14), c.1639-1642del (7), c.2791-2794del (4) suffered both living and kidney dysfunction.

**Table 1:** The variations and clinical information.

**Mutation type**	**Skeletal dysplasia**	**Liver dysfunction**	**Kidney dysfunction**	**Liver and Kidney dysfunction**	**Non-liver or kidney dysfunction**
Missense/Nonsense	27 (52)	23 (52)	8 (52)	7 (52)	28 (52)
Splicing	2 (9)	4 (9)	3 (9)	1 (9)	3 (9)
Small deletions	16 (24)	14 (24)	8 (24)	7 (24)	9 (24)
Small insertion	4 (8)	3 (8)	3 (9)	1 (8)	3 (8)
Small indel	1 (1)	0 (1)	1 (1)	0 (1)	0 (1)
Gross deletion	0 (2)	0 (2)	0 (2)	0 (2)	2 (2)
Total	50 (96)	44 (96)	23 (96)	16 (96)	45 (96)

* *The numbers in brackets represent the total number of the variations*.

However, different patients who had the same mutation may also show different symptoms; for example, the mutation c.560G>A (p.Trp163^*^) was found in a family with two patients; one patient had kidney dysfunction (acute), but the other had none (4). One of the three male patients with mutation c.1290G>A (p.Trp430^*^) from one family showed some epiphyseal dysplasia, but the other two had no obvious epiphyseal dysplasia (14). All these results indicate that there is no specific relationship between the mutation and clinical symptoms.

In this study, we recruited a family including three patients with WRS. WES and Sanger sequencing were performed to identify the mutations that may cause the disease in this family. From the liner structure (5), both of the affected amino acids are located in the kinase domain, indicating that the mutations caused defect on the kinase activity of the PERK. Evolutionary conservation analysis of amino acid residues showed that the amino acid residue Pro940 and Glu994 influenced by the novel mutations are most highly evolutionary conserved among PERK protein from different species, indicating that these mutations are likely pathological.

Because the variable of the clinical phenotype and the difference in gene penetrance except PNDM of the patients with WRS, the diagnosis of WRS was difficult. As the most common gene causing PNDM are *ABCC8* gene and *KCNJ11* gene (9), the analysis of *EIF2AK3* gene is always ignored in molecular diagnosis. It seems that WRS should be considered in the molecular diagnose of PNDM even when the incidence of WRS is very low.

These two mutations in this study were first reported, and it will be helpful for the diagnosis of WRS due to *EIF2AK3* mutations.

## Data Availability Statement

The datasets presented in this study can be found in online repositories. The names of the repository/repositories and accession number(s) can be found below: NCBI [accession: PRJNA719691].

## Ethics Statement

The studies involving human participants were reviewed and approved by Ethics Committees of Shanxi University. Written informed consent to participate in this study was provided by the participants' legal guardian. Written informed consent was obtained from the minor(s)' legal guardian for the publication of any potentially identifiable images or data included in this article.

## Consent for Publication

All patients participating in the study have given written informed consent.

## Author Contributions

YY performed the clinical investigations. NZ participated in the data analysis and drafted the manuscript. NZ carried out the molecular genetic studies. PL and QX helped with the study coordination and proofread the manuscript. CW and HX sponsored, conceived, and designed the study. All authors read and approved the final manuscript.

## Conflict of Interest

The authors declare that the research was conducted in the absence of any commercial or financial relationships that could be construed as a potential conflict of interest.

## References

[1] Wolcott CD Rallison ML. Infancy-onset diabetes mellitus and multiple epiphyseal dysplasia. J Pediatr. (1972) 80:292–7. 10.1016/s0022-3476(72)80596-1 5008828

[2] Delepine M Nicolino M Barrett T Golamaully M Lathrop GM Julier C. EIF2AK3, encoding translation initiation factor 2-alpha kinase 3, is mutated in patients with Wolcott-Rallison syndrome. Nat Genet. (2000) 25:406–9. 10.1038/78085 10932183

[3] Biason-Lauber A Lang-Muritano M Vaccaro T Schoenle EJ. Loss of kinase activity in a patient with Wolcott-Rallison syndrome caused by a novel mutation in the EIF2AK3 gene. Diabetes. (2002) 51:2301–5. 10.2337/diabetes.51.7.2301 12086964

[4] Senée V Vattem KM Delépine M Rainbow LA Haton C Lecoq A. Wolcott-Rallison Syndrome: clinical, genetic, and functional study of EIF2AK3 mutations and suggestion of genetic heterogeneity. Diabetes. (2004) 53:1876–83. 10.2337/diabetes.53.7.1876 15220213

[5] Harding HP Zhang Y Ron D. Protein translation and folding are coupled by an endoplasmic-reticulum-resident kinase. Nature. (1999) 397:271–4. 10.1038/16729 9930704

[6] Harding HP Zhang Y Bertolotti A Zeng H Ron D. Perk is essential for translational regulation and cell survival during the unfolded protein response. Mol Cell. (2000) 5:897–904. 10.1016/s1097-2765(00)80330-5 10882126

[7] Dias RP Buchanan CR Thomas N Lim S Solanki G Connor SE. Os odontoideum in wolcott-rallison syndrome: a case series of 4 patients. Orphanet J Rare Dis. (2016) 11:14. 10.1186/s13023-016-0397-z 26860746 PMC4748609

[8] Asl SN Vakili R Vakili S Soheilipour F Hashemipour M Ghahramani S. Wolcott-Rallison syndrome in Iran: a common cause of neonatal diabetes. J Pediatr Endocrinol Metab. (2019) 32:607–613. 10.1515/jpem-2018-0434 31141482

[9] Welters A Meissner T Konrad K Freiberg C Warncke K Judmaier S. Diabetes management in Wolcott-Rallison syndrome: analysis from the German/Austrian DPV database. Orphanet J Rare Dis. (2020) 15:100. 10.1186/s13023-020-01359-y 32321554 PMC7178620

[10] Xiong Q Li W Li P. Functional evidence for a *de novo* mutation in WDR45 leading to BPAN in a Chinese girl. Mol Genet Genomic Med. (2019) 7:e858. 10.1002/mgg3.858 31332960 PMC6732291

[11] Abecasis GR Auton A Brooks LD DePristo MA Durbin RM Handsaker RE. An integrated map of genetic variation from 1,092 human genomes. Nature. (2012) 491:56–65. 10.1038/nature11632 23128226 PMC3498066

[12] Song W Gardner SA Hayk H Amanda N Katelyn SW Chen WJ. Exploring the landscape of pathogenic genetic variation in the ExAC population database: insights of relevance to variant classification. Genet Med. (2016) 18:850–4. 10.1038/gim.2015.180 26681313

[13] Zhang HJ Wang SB Guo XF Weng B Lin L Hao Y. [A case report of EIF2AK3-related Wolcott-Rallison syndrome and literature review]. Zhongguo Dang Dai Er Ke Za Zhi. (2019) 21:176–9. 10.7499/j.issn.1008-8830.2019.02.014 30782283 PMC7389836

[14] Rubio-Cabezas O Patch AM Minton JA Flanagan SE Edghill EL Hussain K. Wolcott-Rallison syndrome is the most common genetic cause of permanent neonatal diabetes in consanguineous families. J Clin Endocrinol Metab. (2009) 94:4162–70. 10.1210/jc.2009-1137 19837917 PMC2775655

